# Comparison of computer-navigated and conventional total knee arthroplasty in patients with Ranawat type-II valgus deformity: medium-term clinical and radiological results

**DOI:** 10.1186/1471-2474-15-390

**Published:** 2014-11-22

**Authors:** Tsan-Wen Huang, Chien-Yin Lee, Shih-Jie Lin, Kuo-Ti Peng, Kuo-Chin Huang, Mel S Lee, Robert Wen-Wei Hsu, Wun-Jer Shen

**Affiliations:** Department of Orthopaedic Surgery, Chang Gung Memorial Hospital, 6, West Section, Chia-Pu Road, Pu-Tz City, Chia-Yi Hsien 613 Taiwan; Chang Gung University, 259 Wen-Hwa 1st Road, Kwei-Shan Tao-Yuan, 333 Taiwan; Po-Cheng Orthopaedic Institute, 100 Bo-ai 2nd Road, Zuoying District, Kaohsiung Taiwan

**Keywords:** Genu valgus deformity, Computer-navigated surgery, Total knee arthroplasty, Cruciate retaining

## Abstract

**Background:**

Arthritic knees with Ranawat type-II valgus deformity present with soft tissue contracture and osseous anomalies that make total knee arthroplasty (TKA) difficult. We hypothesized that computer-navigated-TKA (CN-TKA) may be superior to conventional techniques and provide better mid-term radiographic and clinical outcomes in such cases.

**Methods:**

Between January 2002 and January 2009, patients with Ranawat type-II valgus deformity who underwent primary TKA were entered into this retrospective study. Conventional TKA and CN-TKA were compared for the accuracy of component placement, joint line level, and postoperative limb alignment. International Knee Society scores and patellar scores were used for clinical assessment.

**Results:**

A total of 62 patients (70 knees) with a minimum of 5 years of follow-up were studied. Conventional TKA was performed in 36 knees and CN-TKA in 34 knees. A significantly higher rate of lateral retinaculum release was recorded in the conventional TKA group compared to the CN-TKA group. Proper restoration of joint line was achieved using CN-TKA. The range of motion of the knees was similar in both groups preoperative and postoperatively. There were no significant differences in reconstructed mechanical axes, accuracy of component positioning, and difference in perioperative hemoglobin level between the two groups. At a mean follow-up of 6.2 years, both groups had significant postoperative improvements in clinical performance, however the difference did not reach statistical significance between both techniques.

**Conclusions:**

Our findings demonstrate that CN-TKA can properly restored the joint line level for arthritic knees with Ranawat type II valgus deformity. However, no differences in clinical function, limb and component alignment, or survival of the prostheses were noted between the CN-TKA and conventional TKA groups at a mean follow-up of 6.2 years.

**Electronic supplementary material:**

The online version of this article (doi:10.1186/1471-2474-15-390) contains supplementary material, which is available to authorized users.

## Background

Total knee arthroplasty (TKA) is a successful procedure for end-stage arthritis of the knee joint [1]. Studies on the biomechanical and long-term survival have reported that the reconstructed mechanical axis and component positions should be within 3 degrees of neutral to prevent abnormal wear, premature mechanical loosening of the components, and patellofemoral problems [2–5]. Following the development of computer-navigated TKA (CN-TKA), more accurate bone cuts, more precise component placement in coronal, sagittal and axial planes, better restoration of coronal limb alignment and joint line, and less gap asymmetry have been reported [6–13].

Approximately 10% of patients requiring TKA have a valgus deformity, and this deformity was classified into three types by Ranawat et al. [14, 15]. The type-I deformity accounts for 80% of all valgus knees, and is defined as a preoperative valgus angulation of anatomical axis <10° based on hip-to-ankle standing radiography. The medial collateral ligament (MCL) is functional and intact in this type of deformity, and performing TKA is not more challenging than usual. The type-III deformity (5% of valgus knees) includes an anatomical axis >20° and an impaired medial soft-tissue sleeve. A varus-valgus constrained type of prosthesis is often required to compensate for the lax MCL. The type-II valgus deformity is seen in 15% of valgus knees, and has a more substantial deformity (anatomical axis ≥10°) with medial soft tissue being stretched and elongated, but functional. Furthermore, the osseous deformity in Ranawat type-II valgus deformity (including hypoplasia of the distal femur, rotational deformity of the tibia and femur, unusual proximal femoral neck-shaft angles, and maltracking of the patella) may influence the accuracy of conventional instrumentation and contribute to improper bone cuts. In this circumstance, conventional TKA is technically challenging and may have greater risks of malposition of the components, improper joint line and unplanned conversion to a constrained prosthesis intraoperatively [14–16]. Some surgeons find it difficult to correct an advanced valgus deformity using conventional instrumentation without a constrained prosthesis [17–19].

CN-TKA has the theoretical advantages in dealing with Ranawat type-II valgus deformity of precise bone cuts in conjunction with meticulous soft tissue release with real-time and quantitative feedback [20]. However, only a few cohort studies have been published regarding whether or not radiographic improvements achieved with CN-TKA in valgus deformity lead to an improvement in functional scores or implant survival after mid- or long-term follow-up [21, 22]. We hypothesized that CN-TKA would be superior to conventional TKA, and thus the aim of this study was to retrospectively investigate the effect of CN-TKA compared with conventional TKA on mid-term clinical function, alignment, and implant survival.

## Methods

### Demographics

Between January 2002 and January 2009, patients who had arthritic knees with Ranawat type-II valgus deformity (a preoperative valgus angulation of anatomical axis ≥10° based on hip-to-ankle standing radiography) and who underwent primary TKA were enrolled in this retrospective study. The cost of CN was not reimbursed in our national health insurance, the type of surgery was chosen by the patients themselves after well explaining the merits of both techniques to patients.

The clinical and radiographic data and functional outcomes were reviewed. The clinical data included age, gender, diagnosis, intraoperative procedures, tourniquet time (the tourniquet was inflated before skin incision and was deflated after the setting of cement), difference in perioperative hemoglobin level (which was checked before the operation and in the morning of the second postoperative day), and antibiotic prophylaxis. The radiographic and functional assessments before surgery and at the time of each follow-up were analyzed and recorded by a research associate (Yu-Shuan, Lin) who was not part of the operative team and was blinded to technique allocation. This study was approved by the Ethics Committee and Institutional Review Board of Chang Gung Memorial Hospital (99-2386B). Informed consent for participation in the study was obtained from all participants.

### Surgical technique

A tourniquet was used for all patients. The procedure was carried out through a midline skin incision with the use of a medial parapatellar arthrotomy. All cases received the same cruciate-retaining type of cemented total knee prosthesis (DePuy PFC Knee Systems, DePuy Orthopaedics, Warsaw, Indiana).

**Figure 1:**
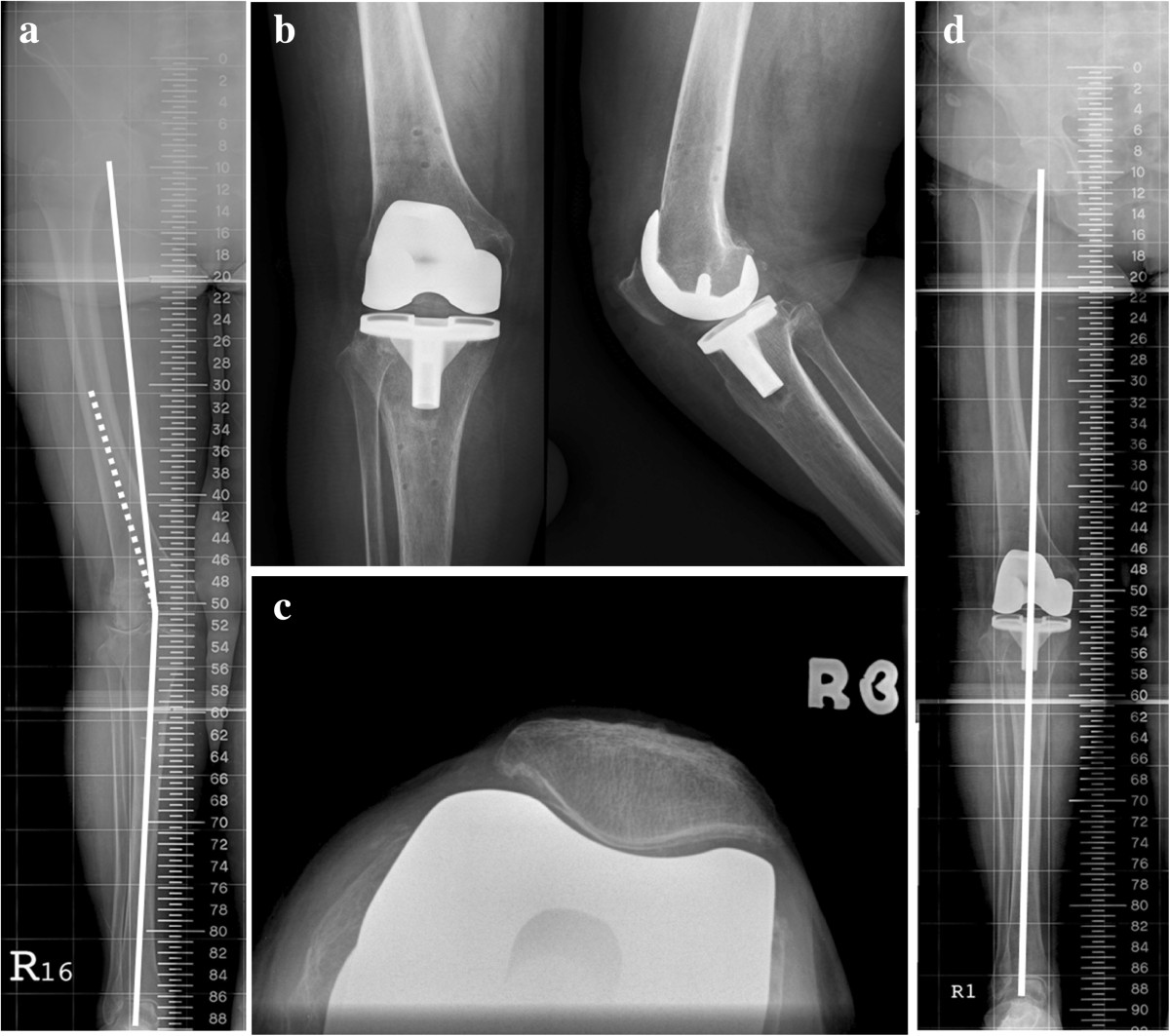
**A 74-year female with advanced valgus deformity of the right knee joint. a**. The preoperative hip-to-ankle standing radiography showing significant genu valgus deformity with preoperative anatomical axis of 16.1°. **b**. Radiograph after total knee replacement with a cruciate-retaining type prosthesis. **c**. Radiograph of post-operative patella axial view after total knee replacement with a cruciate-retaining type prosthesis. **d**. Postoperative radiograph showing complete restoration of limb alignment after undergoing CN-TKA.

In all of the conventional TKA procedures, the prosthesis was implanted using an intramedullary guidance system for the femoral component and an extramedullary guide for the tibial side. After removal of osteophytes from the femur, the soft tissues of the lateral compartment were then released from the proximal tibia. The distal femoral cut was based on the valgus correction angle of the distal femur (measured on hip-to-ankle standing radiography [23]) (Figure 1). Instead of 3 degrees of external rotation relative to the posterior aspect of the condyles, femoral component rotational alignment was determined according to the transepicondylar axis. The Whiteside’s line, posterior condylar line and the tibial cutting plane were supplementary to judge the femoral rotation. A spacer block was then used to check the rectangular gap in flexion and extension. The soft tissue balance was assessed at trial reduction and achieved by sequential release of the tight structures (iliotibial band, popliteus, lateral collateral ligament, and the lateral head of the gastrocnemius) as recommended by Whiteside [24]. The posterior cruciate ligament (PCL) was assessed using the pull-out lift-off test reported by Scott and Chmell [25], and the PCL was then recessed as needed from its insertion site in the tibia to obtain the desired tension. The assessment of bony defect are concerning because of the potential for later mechanical failure. It is important to determine the extent of the bony defect, the integrity of the cortex, and the stability of the component fixation. If the bone loss is minimal and the cortical rim is intact, we addressed the small cavitary defects with use of bone cement or cancellous bone chips. For patients with greater bone loss with poor integrity of cortical rim, structural allografts were applied to for restoration of the proximal part of the tibia or the distal part of the femur. After the cement had fully set, the tourniquet was then deflated and hemostasis. Assessment of patellar tracking were performed. The indication for release the lateral retinaculum is according to “The no thumb test”.

The CN-TKA was implanted with the use of a computed tomography (CT)-free navigation system (BrainLAB, Munich, Germany). After exposing the knee joint by using an anterior midline longitudinal skin incision and a medial parapatellar arthrotomy, the synovium was adequately removed to make registration precise. The femoral reference arrays were fixed to the distal femur using two bi-cortical half-pins, and the tibial reference arrays were fixed to the proximal tibia. An infrared camera was used to track the femoral and tibial reference arrays. The implant size and orientation were identified by dragging the pointer along the bone surface to reconstruct the three-dimensional bone model. The rotational alignment of the femur was checked by the transepicondylar axis under navigation. The contracted soft tissue was sequentially released using Whiteside’s method [24], and the tension of the PCL was assessed using the method of Scott and Chmell [25] with real-time and quantitative feedback of the navigation. The assessment and management of bony defect were performed. The femoral and tibial reference arrays were retained until the cement had fully set, and were removed after the alignment was verified under navigation. The tourniquet was then deflated and hemostasis. Assessment of patellar tracking were performed by using “The no thumb test”. All procedures were performed by a senior surgeon (Hsu RWW) who has extensive experience in the use of both conventional mechanical guides and computer-assisted navigation.

### Rehabilitation

A continuous passive motion machine was used for passive range of motion exercises four times daily (30 minutes per exercise period) from the day of surgery until the day of discharge from the hospital. Under the supervision of a physical therapist, the patients started active knee-motion exercises and began standing at the bedside or walking with crutches or a walker twice daily for 30-minute periods. All patients used crutches or a walker with full weight-bearing for six weeks, and a cane when needed thereafter.

### Radiographic evaluation

All patients received hip-to-ankle standing radiography along with anteroposterior and lateral radiography of the knees and the skyline view of the patellofemoral joint before and after surgery, following a protocol described in detail in a previous publication by the senior author [26]. The lateral patellar tilt and displacement were measured according to the method of Laurin et al. [27, 28]. The postoperative patellar tilting angle was measured by the method described by Kawahara S et al. [29]. Radiographic parameters including the mechanical axis and four component alignment angles (the femoral valgus angle, tibial valgus angle, femoral flexion angle, and tibial flexion angle [30]) were assessed. The position of the prosthetic joint line was measured on radiographs taken before surgery and at the last follow-up using the method proposed by Figgie et al. [31]. In brief, the length of the joint line measured in the lateral view was the perpendicular distance from a weight bearing surface of the tibial plateau to the tibial tubercle of the natural tibia. The relative change in joint line distance was recorded. All measurements were done by a blinded observer using digital radiographs.

We considered a tibial component positioned at a valgus angle of 90° in the coronal plane and at a flexion angle of 87° in the sagittal plane (3° of posterior slope) to be ideal. A femoral component positioned at a flexion angle of 0° in the sagittal plane was considered to be ideal, and the desired valgus angle in the coronal plane was determined according to the valgus correction angle of the distal femur which was measured by hip-to-ankle standing radiography. The goal of alignment was to correct the postoperative mechanical axis to within 3° of neutral, and all component angles to within 3 degrees of the ideal.

### Clinical evaluation

Preoperative and postoperative functional scores were calculated using the International Knee Society (IKS) scoring system [32] and patellar score [33]. The patellar score was designed by Feller et al. to assess the management of the patellofemoral joint. Active maximum range of motion of the knee was measured using a goniometer. The patients who had extra-articular deformity of the femur or tibia related to trauma or previous surgery and those with incomplete medical records, radiographic analysis and functional evaluation were excluded.

### Statistical analysis

All data were entered into an Excel spreadsheet (Microsoft Corp, Redmond, WA), checked for missing and illogical entries, and subsequently copied into SPSS version 13.0 (SPSS Inc., Chicago, IL). Statistical analysis was performed by an independent statistician blinded to the surgical procedures. The independent *t* test and chi-square test were used for comparisons of preoperative and postoperative (the last follow-up visit) radiographic parameters and functional results. The level of statistical significance was set at p <0.05.

## Results

### Clinical data

A total of 62 patients (70 knees) were enrolled in this study, with a mean age of 71.3 years (range, 50 to 86 years) at the time of surgery. The diagnosis was degenerative osteoarthritis in 68 knees and rheumatoid arthritis in the other two. The mean body height was 156.2 cm (range, 133.0 to 174.0 cm), mean body weight 64.8 kg (range, 35.0 to 95.0 kg), and mean body mass index 27.5 kg/m^2^ (range, 19.8 to 38.1 kg/m^2^). The mean follow-up time was 6.2 years (range, 5–11 years).

Thirty-six knees underwent conventional TKA and thirty-four knees underwent CN-TKA. There were no statistically significant differences between the two groups with regards to age at the time of surgery, body height, body weight, body mass index, duration of hospital stay, and length of follow-up (Table 1).

**Table 1:** **Demographic data of the patients in the conventional and CN-TKA groups**

Parameters	Group A	Group B	
	N = 36	N = 34	***p***-value
**Age (years)**	70.6 (53–86)	70.3 (50–85)	0.895
**Operation site**			0.332
**Right**	22	24	
**Left**	14	10	
**Body height (cm)**	155.6 (147.0 – 171.5)	154.4 (133.0 – 174.0)	0.716
**Body weight (kg)**	65.7 (47.2 – 79.1)	66.5 (35.4 – 95.3)	0.504
**Body mass index (kg/m** ^**2**^ **)**	26.8 (21.8 – 31.1)	27.1 (19.8 – 38.1)	0.324
**Reason for surgery**			1.000
Primary osteoarthritis	35 (97.2 %)	33 (97.1 %)	
Rheumatoid arthritis	1 (2.8 %)	1 (2.9 %)	
**Hospital stay (days)**	6.8 (5–10)	6.3 (4–10)	0.114
**Follow-up time (years)**	6.7 (5–11)	6.1 (5–10)	0.148

Group A: Knees undergoing conventional TKA.

Group B: Knees undergoing CN-TKA.

The values are given as the mean with the range in parentheses or (%) where appropriate.

*P*-values for between-group comparison were determined by chi-square tests for categorical variables, and the t test for continuous variables.

*Statistically significant (*p* value <0.05).

The difference in perioperative hemoglobin level was less in the CN-TKA group than in the conventional TKA group, however the difference did not reach statistical significance. No significant difference in tourniquet time was noted. Theoretically, the reference base attachments and registration should have led to a longer tourniquet time in the CN-TKA cohort. However, intraoperatively repeated assessments of the position of the femoral component according to multiple reference lines in the conventional cohort may have also resulted in a longer tourniquet time. Thirteen knees (36.1%) in the conventional TKA group required release of the lateral retinaculum to obtain adequate patellar tracking, while only 2 knees (5.9%) required release in the CN-TKA group (*p* = 0.001). Bone grafting was performed in 7 knees (19.4%) in the conventional TKA group and 11 knees (32.4%) in the CN-TKA group (Table 2).

**Table 2:** **Perioperative and radiographic data of the conventional and CN-TKA groups**

Parameters	Group A	Group B	***p***-value
	N = 36	N = 34	
***Perioperative data***			
**Difference of perioperative hemoglobin level (g/dL)**	1.5 ( 0.6–2.7)	1.3 (0.4–2.1)	0.448
**Tourniquet time (minutes)**	75 (40–149)	79 (58–117)	0.532
**Lateral retinaculum for patellar tracking**	13 (36.1%)	2 (5.9%)	0.001*
**Bone grafting**	7 (19.4%)	11 (32.4%)	0. 086
***Radiographic data***			
**Preoperative AA (deg)**	195.4° (191°−204°)	194.3° (192°−202°)	0.202
**Postoperative MA (deg)**	179.9° (176°−184°)	180.4° (178°−181°)	0.307
**Preoperative congruent angle (deg)**	12.6° (1°−39°	11.5° (2°−38°)	0.716
**Postoperative patellar tilting angle (deg)**	2.7 (2°−5°)	2.4° (range, 1°−4°)	0.855
**Joint line elevation (mm)**	2.24 (−1.9−6.2)	1.33 (−1.3−2.7)	0.020*
**Component alignment**			
Femoral valgus angle (deg)	95.4° (93°−97°)	94.6° (92°−96°)	0.448
Femoral flexion angle (deg)	3.1° (0°−7°)	2.7° (0°−7°)	0.165
Tibial valgus angle (deg)	90.1° (89°−91°)	89.9° (88°−91°)	0.960
Tibial flexion angle (deg)	86.6° (83°−91°)	87.1° (84°−90°)	0.573

Group A: Knees undergoing conventional TKA.

Group B: Knees undergoing CN-TKA.

AA = anatomic axis, MA = mechanical axis.

The values are given as the mean with the range in parentheses or (%) where appropriate.

*P* values for between-group comparison were determined by chi-square tests for categorical variables, and the t test for continuous variables.

*Statistically significant (*p* value <0.05).

### Radiographic results

In the radiographic evaluation, we found no differences between the two groups in preoperative anatomical axis and postoperative mechanical axis. The mean preoperative anatomical axis was 195.4° (range, 191°-204°) in the conventional TKA group and 194.3° (range, 192°-202°) in the CN-TKA group. The postoperative mechanical axis was corrected to 179.9° (range, 176°-184°) in the conventional TKA group and 180.4° (range, 178°-181°) in the CN-TKA group. The mean preoperative patellofemoral congruence angles were 12.6° (range, 1°-39°) in group A and 11.5° (range, 2°-38°) in group B. There was no statistical significance (*p* = 0.716). The postoperative patellar component-tilting angle was 2.7 (range, 2°-5°) in group and 2.4° (range, 1°-4°) in group B. There was still no statistical significance (*p* = 0.855)”. There was less joint line elevation in the CN-TKA group than the conventional TKA group (*p* = 0.020). No postoperative differences were noted between the two groups with regards to the four component alignment angles including femoral valgus angle (*p* = 0.448), femoral flexion angle (*p* = 0.165), tibial valgus angle (*p* = 0.960) and tibial flexion angle (*p* = 0.573) (Table 2). The percentage of procedures achieving the ideal reconstructed mechanical axis and component alignments were similar between the two groups (Table 3).

**Table 3:** **Comparison of percentage of postoperative lower limb axis (within 3° deviation) and component positioning between the conventional and CN-TKA groups**

	No. of postoperative component positioning within 3°deviation		
	Group A	Group B	***p***-value
	N = 36	N = 34	
**Mechanical axis within 3° deviation**	30 (83.3%)	32 (94.1%)	0.267
**Component positioning**			
Femoral valgus angle	35 (97.2%)	34 (100%)	1.000
Femoral flexion angle	20 (55.6%)	26 (76.5%)	0.136
Tibial valgus angle	36 (100%)	34 (100%)	-
Tibial flexion angle	33 (91.7%)	31 (91.2%)	1.000

Group A: Knees undergoing conventional TKA.

Group B: Knees undergoing CN-TKA.

The values are given as the n (%).

*P* values for between-group comparison were determined by chi-square tests.

*Statistically significant (*p* value <0.05).

### Functional results

Clinically, the active range of motion improved from 97.6° to 113.3° in the conventional TKA group, and from 96.9° to 114.3° in the CN-TKA group. The mean patellar score improved markedly after surgery in both groups (from 17.3 to 26.2 in the conventional TKA group, and from 16.4 to 26.7 in the CN-TKA group). On the IKS scoring system, the mean pain score improved from 16.4 to 47.4 in the conventional TKA group, and from 14.3 to 48.1 in the CN-TKA group, the mean clinical knee score improved from 40.5 to 95.2, and from 39.3 to 96.7, respectively, and the mean functional knee score improved from 35.3 to 94.6, and from 33.4 to 96.3, respectively (Table 4).

**Table 4:** **Comparison of preoperative and postoperative knee scores between the conventional and CN-TKA groups**

	Group A	Group B	***p***-value
	N = 36	N = 34	
***Preoperative functional score***			
Patellar score (points)	17.3 (10–24)	16.4 (10–24)	0.541
IKS score for pain (points)	16.4 (10–20)	14.3 (10–20)	0.084
IKS score for clinical knee score (points)	40.5 (11–65)	39.3 (16–60)	0.668
IKS score for functional knee score (points)	35.3 (20–55)	33.4 (20–50)	0.445
Active range of motion in degrees (deg)	97.6 (50°−120°)	96.9 (80°−120°)	0.822
***Postoperative functional score***			
Patellar score (points)	26.2 (20–30)	26.7 (21–30)	0.635
IKS score for pain (points)	47.4 (40–50)	48.1 (40–50)	0.315
IKS score for clinical knee score (points)	95.2 (87–100)	96.7 (90–100)	0.102
IKS score for functional knee score (points)	94.6 (80–100)	96.3 (90–100)	0.217
Active range of motion in degrees (deg)	113.3 (100°−125°)	114.3 (105°−125°)	0.583

Group A: Knees undergoing conventional TKA.

Group B: Knees undergoing CN-TKA.

IKS Score = International Knee Society Score.

The values are given as the mean with the range in parentheses.

*P* values for between-group comparison were determined by t tests.

*Statistically significant (*p* value <0.05).

### Complications

No patient required advancement of MCL or was converted to a posterior-stabilized prosthesis or varus-valgus constrained components because of excessive bony resection or inadequate soft tissue release during the operation. There were no cases of wound infection, peroneal nerve neurapraxia, pulmonary emboli, or deep vein thrombosis. No periprosthetic fractures, joint instability, or patellar tracking problems were encountered. No patients showed loosening or osteolysis on radiography at the time of the last follow-up, and no patients received revision surgery for any reason.

## Discussion

Proper alignment of limb and component positions as well as soft tissue balance during TKA is critical in maximizing implant survival [2–5]. It has been reported that CN-TKA allows for a higher accuracy in radiographic parameters and soft tissue balance [6–13]. The osseous deformity in Ranawat type-II valgus deformity may influence the accuracy of conventional instrumentation and contribute to improper bone cuts [14–19]. However, compared to conventional instrumentation, CN-TKA only focuses on the center of the hip, knee, and ankle joints and overlooks extra-articular deformities of the femur and tibia. In addition, CN-TKA provides real-time and quantitative feedback to the surgeon [20]. These characteristics are theoretically beneficial for Ranawat type-II valgus deformities.

A few studies have shown that CN-TKA improved the overall accuracy of limb alignment and positioning of components in patients with valgus deformities [21, 22]. Hadjicostas et al. [21] studied 15 patients with valgus deformity of 17°-27°, and reported excellent mid-term results using CN-TKA combined with simultaneous osteotomy of the lateral femoral condyle. Chou et al. [22] reported a case-series study with a mean follow-up of 37.8 months, and concluded that CN-TKA in conjunction with preoperative radiographic templating is an alternative strategy to improve radiographic and clinical results in arthritic valgus knees. However, studies to confirm that the improved radiographic alignment achieved with CN-TKA translates into better mid- or long-term clinical outcomes or the durability of the TKA for Ranawat type-II valgus deformities are lacking. In addition, previous reports have been limited by small sample size, different severity of valgus deformity, short follow-up, and lack of a control group for comparisons to demonstrate the usefulness of CN-TKA. In the current study, CN-TKA provided proper radiographic and functional results without the need for additional osteotomy, and no significant differences with regards to limb alignment, component position and functional outcomes were noted between both techniques even at a mean follow-up of 6.2 years. The absence of significant differences in outliers in radiographic parameters may explain these results.

In TKA, restoration of the joint line is a crucial objective, as an incorrect joint line level may contribute to joint instability, anterior knee pain, limited range of motion, joint stiffness, patellofemoral contact forces, acceleration of polyethylene wear, and inferior clinical results [22, 31, 34, 35]. In order to obtain adequate stability of the knee joint, a thicker tibial polyethylene or unplanned conversion to a varus-valgus constrained type of prosthesis is necessary to compensate for improper bony resection and excessive soft tissue release. However, thicker tibial polyethylene would also result in elevation of the joint line level and increase the risk of peroneal nerve neurapraxia. Chou et al. [22] reported that the joint line was well restored with the use of navigation. Ensini et al. [35] concluded that a navigation system can support intraoperative joint line restoration by controlling the exact resection of the bone and the corresponding overall thickness of the components implanted. In the current study, we found a statistically significant difference in joint line elevation between CN-TKA and conventional TKA (*p* = 0.020) (Table 2).

In the current study, 13 knees (36.1%) in the conventional TKA group required release of the lateral retinaculum to obtain adequate patellar tracking, while only 2 knees (5.9%) required release in the CN-TKA group (*p* = 0.001). When using conventional femoral guidance jigs, deficiencies of the lateral femoral condyle often render the posterior condylar axis unreliable as a reference to determine femoral component rotation, resulting in an inadequately rotated femoral component. Malrotation of the femoral component affects patellofemoral tracking and may increase the contact pressure and lead to accelerated and excessive wear of the patellar button [36, 37].

Reliable evidence of the effect of CN-TKA on improvements in femoral rotational alignment and implant survival is limited because intraoperative and postoperative measurement techniques are often inaccurate, and because the optimal rotational alignment target has not been defined [13]. Using postoperative CT scans, several authors have demonstrated significant improvements in the rotational alignment of the femoral component with CN-TKA [6, 38–40], however, other researchers have reported no differences between CN-TKA and conventional TKA [13, 41]. This discrepancy may reflect, at least in part, the different types of preoperative knee deformities (varus or valgus deformity) seen in their respective study populations. Further studies using CT to evaluate the rotational alignment are necessary to determine whether CN-TKA merits alternative use in conventional TKA in genu valgum or genu varum deformities.

There are several strengths to this study. First, despite the relative rarity of Ranawat type-II valgus deformity (approximately 10% of patients requiring TKA have a valgus deformity, and type-II deformity accounts for 15% of all valgus knees), we conducted a relatively large-scale cohort study (36 TKAs implanted using conventional instrumentation and 34 using CN-TKA) to compare the alignment and mid-term clinical outcomes. Second, all patients underwent surgery by a single surgeon, the single prosthesis used, the same surgical technique, and the same protocol allowed for decreases in confounding factors.

Several limitations to this study should be acknowledged. First, this was a retrospective study with all its inherent limitations and bias. Second, because the cost of CN was not reimbursed in our national health insurance, the type of surgery was chosen by the patients themselves based on their socioeconomic status. This potential bias may be related to outcome. Third, this study is limited by mid-term clinical follow-up, and we were unable to assess whether proper alignment would translate into long-term functional outcomes. Finally, a significantly higher rate of lateral retinaculum release was recorded in the conventional TKA group. However, both groups had similar patellar scores, and we are unable to state for certain whether CN-TKA would produce better benefits in this situation. We were also unable to provide data regarding rotational alignment of the femoral components. Further studies using CT to evaluate the rotational alignment are necessary to determine whether CN-TKA merits alternative use in conventional TKA.

## Conclusion

CN-TKA is theoretically useful for arthritic knees with Ranawat type II valgus deformity where accurate restoration of joint line, proper alignment of limb and component positioning may be challenging because of associated osseous deformities and contracture of soft tissue. Our results demonstrated that CN-TKA can provide proper restoration of the joint line level, however there were no differences in clinical function, limb and component alignment, as well as survival of the prosthesis between the CN-TKA and conventional TKA groups after a mean follow-up of 6.2 years.

## Authors’ original submitted files for images

Below are the links to the authors’ original submitted files for images. Authors’ original file for figure 1 Authors’ original file for figure 2 Authors’ original file for figure 3 Authors’ original file for figure 4
