# Supplementary figures and images for: Comparison of computer-navigated and conventional total knee arthroplasty in patients with Ranawat type-II valgus deformity: medium-term clinical and radiological results
Source: BMC Musculoskelet Disord. 2014 Nov 22;15:390. doi: 10.1186/1471-2474-15-390 (PMC4258048; doi:10.1186/1471-2474-15-390)

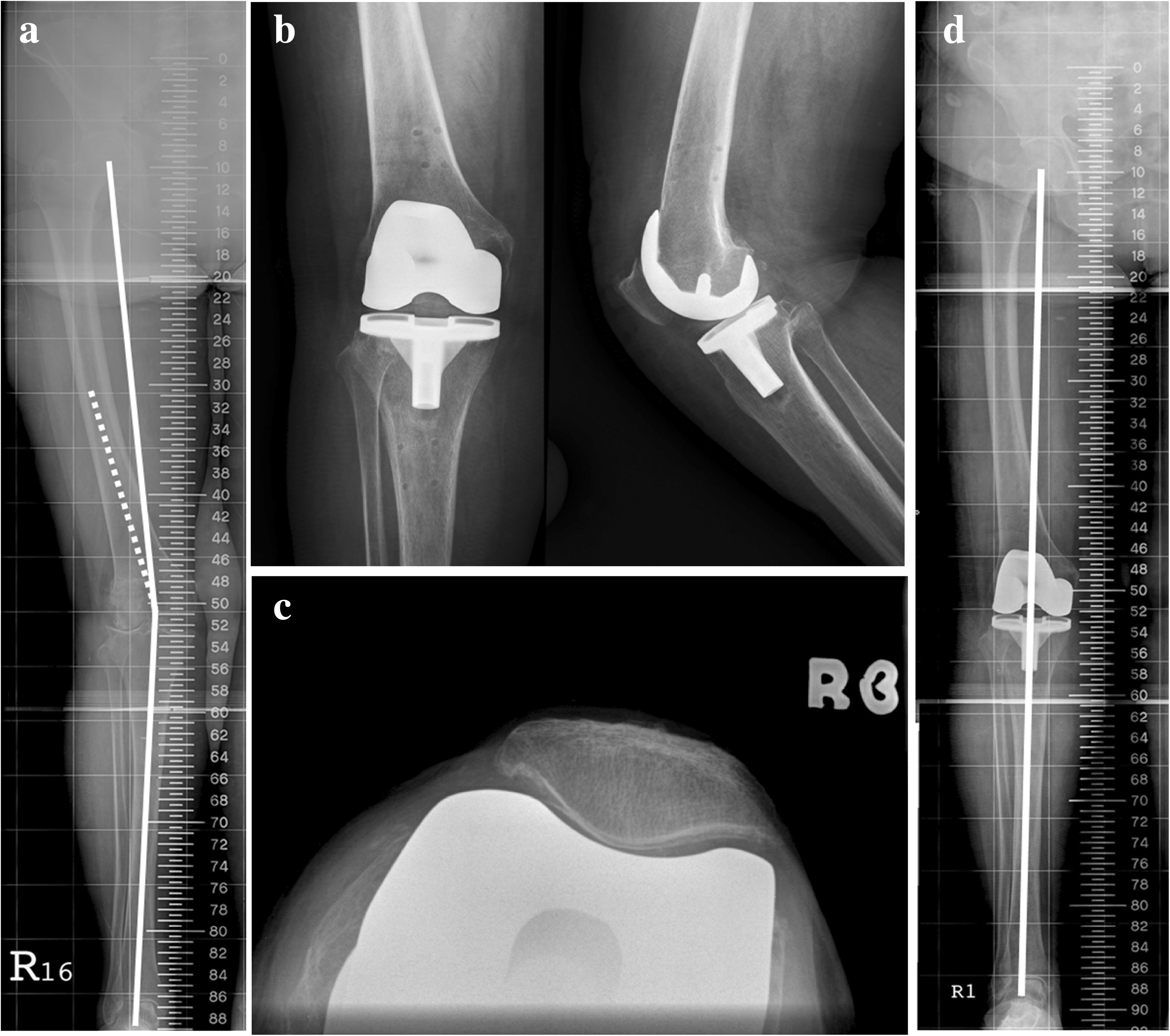

Supplement: Supplementary file 1 — Authors’ original file for figure 1 [file 12891_2014_2338_MOESM1_ESM.tif]

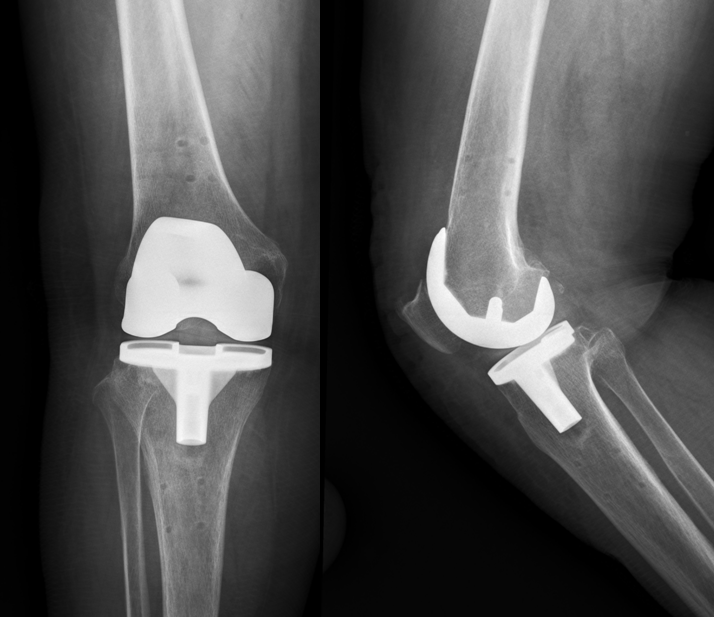

Supplement: Supplementary file 2 — Authors’ original file for figure 2 [file 12891_2014_2338_MOESM2_ESM.tiff]

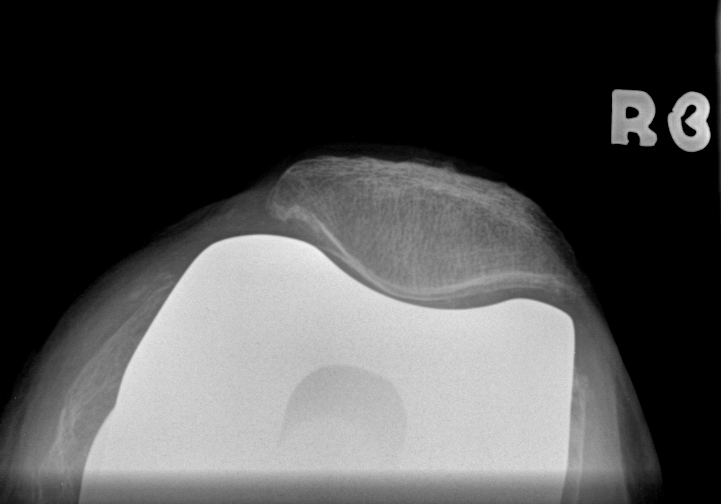

Supplement: Supplementary file 3 — Authors’ original file for figure 3 [file 12891_2014_2338_MOESM3_ESM.tiff]

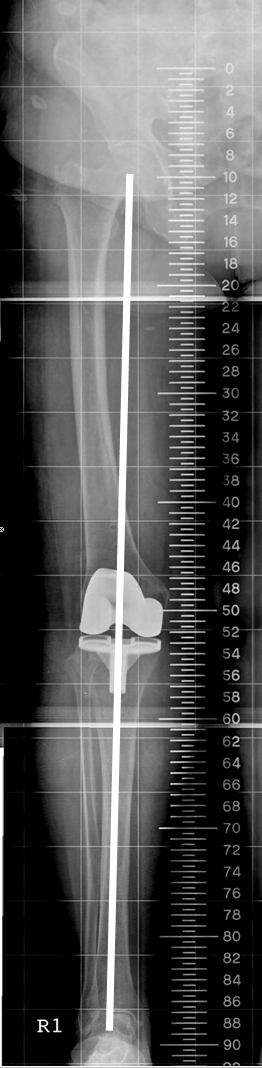

Supplement: Supplementary file 4 — Authors’ original file for figure 4 [file 12891_2014_2338_MOESM4_ESM.tiff]
